# Spatiotemporal patterns and 2025 forecasting of other infectious diarrheal diseases in Beijing, China: a 21-year population-based study (2004-2024)

**DOI:** 10.1186/s12889-026-27967-0

**Published:** 2026-06-04

**Authors:** Jiaxin Feng, Baiwei Liu, Xiaona Wu, Li Zhang, Shuyu Ni, Dan Du, Zhaomin Feng, Peng Yang, Quanyi Wang, Zhiyong Gao, Daitao Zhang

**Affiliations:** 1 Beijing Key Laboratory of Surveillance, Early Warning and Pathogen Research On Emerging Infectious Diseases, Beijing Center for Disease Prevention and Control, No. 16 Hepingli Middle Street, Dongcheng District, Beijing, 100013 China; 2 School of Public Health, Capital Medical University, Beijing, 100069 China

**Keywords:** Other infectious diarrheal diseases, Descriptive epidemiology, Spatiotemporal distribution, SARIMA model, Incidence forecast

## Abstract

**Background:**

Other Infectious Diarrheal Diseases (OIDD), caused by pathogens excluding *Vibrio cholerae, Shigella, Salmonella typhi*, and *Salmonella paratyphi*, remain a significant public health concern in China. Beijing, a megacity with high population density, extensive mobility, and a temperate monsoon climate, is a prototypical example of temperate monsoon megacities globally. Long-term OIDD patterns, spatiotemporal distribution, and future trends in such dense urban settings are poorly understood, and insights from Beijing can provide valuable guidance for similar cities worldwide.

**Methods:**

We retrieved case data on OIDD reported in Beijing during 2004-2024 from the Chinese Disease Prevention and Control Information System. Descriptive epidemiology, spatial autocorrelation analysis, hotspot analysis, and the Seasonal Autoregressive Integrated Moving Average (SARIMA) model were employed to investigate the temporal, demographic and regional distribution patterns, identify high-risk regions, and forecast the incidence of OIDD for 2025.

**Results:**

A total of 756,933 OIDD cases were reported over the 21-year period. Incidence exhibited an overall downward trend from 2006 to 2022, followed by a rebound from 2023 to 2024. Seasonal peaks occurred in summer (July-August) and winter (December-January), with a notable shift of the summer peak to April-May in 2023. The under-1 year age group showed the highest average annual incidence rate (3502.75 per 100,000), and males were more affected than females (male-to-female ratio 1.19:1). Spatial analysis revealed significant clustering (Moran’s *I* = 0.18-0.44, *P* < 0.01), with high-high clusters shifting from central urban districts to suburban areas over time, while persistent low-low clusters were observed in remote districts. The SARIMA (1,1,1) (1,1,1) ₁₂ model accurately predicted monthly cases in 2024 (MAPE = 17.46%) and forecast 32,638 OIDD cases in 2025, following a bimodal seasonal pattern.

**Conclusions:**

This study depicted the 21‑year spatiotemporal epidemiological characteristics of OIDD in Beijing and validated a predictive SARIMA model for future incidence. OIDD showed a downward trend followed by a recent rebound, obvious bimodal seasonality, a heavy burden among young children, and shifting spatial clusters. These results provide key evidence for targeting suburban areas, young children, and seasonal peaks in prevention strategies.

**Supplementary Information:**

The online version contains supplementary material available at https://doi.org/10.1186/s12889-026-27967-0.

## Introduction

Other Infectious Diarrhea Diseases (OIDD), defined as intestinal infections caused by pathogens other than *Vibrio cholerae*, *Shigella*, *Salmonella typhi*, and *Salmonella paratyphi*, is classified as Class C notifiable infectious diseases in China [1]. The incidence of these diseases remains persistently high nationwide. According to the 2024 National Notifiable Infectious Disease Surveillance Report, more than 1.5 million OIDD cases were reported nationwide, ranking second among all Class C notifiable diseases, with young children being particularly vulnerable [2].

Beijing, as a megacity with over 20 million residents, high population density, frequent domestic and international mobility, a complex urban environment, and a temperate monsoon climate, represents a unique epidemiological setting where transmission risks are elevated. Notably, OIDD epidemiology differs between northern and southern China: southern Fujian, a coastal province, has a persistently high incidence, with seasonal peaks shifting from autumn to winter and spring [3]. In contrast, northern megacities like Beijing have unique epidemiological features shaped by their temperate monsoon climate and dense urbanization, yet long-term, citywide analyses of OIDD incidence, spatiotemporal clustering, and predictive modeling remain limited. Existing studies often cover short observation periods, have low spatial resolution, or lack forecasting components, which restricts their utility for early warning and targeted interventions [4, 5].

Since the establishment of the China Disease Prevention and Control Information System in 2004, consistently high-quality surveillance data have been accumulated. Utilizing surveillance data from 2004 to 2024, this study integrates descriptive epidemiological analysis, spatial epidemiology methods, and time series forecasting models to characterize the long-term epidemiological trends of OIDD in Beijing, elucidate its spatiotemporal clustering patterns, and project the incidence trend for 2025. The findings will provide a scientific basis for early warning and targeted prevention and control strategies in Beijing, and serve as a reference for infectious disease management in other densely populated metropolitan areas.

## Methods

### Data sources and case definition

This study collected data on cases of OIDD reported in Beijing between January 1, 2004, and December 31, 2024, through the China Disease Prevention and Control Information System (the national infectious disease surveillance system). The statistical timeline was based on the date of symptom onset. Demographic data were obtained from district-level statistical bureaus of Beijing. Geographic data were sourced from the National Geographic Information Resource Directory Service, with a vector map of Beijing at the township/subdistrict level (scale: 1:1,000,000) serving as the base map.

OIDD is defined according to the Chinese national diagnostic criteria for infectious diarrhea (WS 271-2007). OIDD refers to a group of intestinal infectious diseases caused by pathogens other than *Vibrio cholerae*, *Shigella*, *Salmonella typhi* and *Salmonella paratyphi*, with diarrhea as the main clinical feature. The diagnostic criteria are divided into clinically diagnosed cases and confirmed cases, as per the national diagnostic standard. Clinically diagnosed cases refer to those with bowel movements of more than three times a day, abnormal stool consistency, a usually short course of disease, clear evidence of infection, a history of unclean diet (water), contact with patients with diarrhea, or recent travel history, etc. Confirmed cases meet the above clinical diagnostic criteria for cases, and the microbiological examination of fecal specimens indicates a positive pathogen, or a positive specific antigen or nucleic acid.

### Epidemiological and temporal analysis

This initial analytical step aimed to establish the baseline epidemiological profile of OIDD in Beijing over the 21-year study period. These descriptive findings provided the foundation for subsequent spatial and predictive analyses.

Descriptive epidemiological methods were employed to systematically analyze the epidemiological characteristics of OIDD in Beijing from 2004 to 2024 across temporal, demographic and regional dimensions. Incidence trends were assessed using the Cochran-Armitage trend test (a chi-square test for trend), with the annual incidence rate as the response variable and calendar year as the ordered exposure. The test statistic follows a chi-square distribution with one degree of freedom. Seasonal indices were calculated based on a moving-average time series decomposition method, and seasonal strength was computed for each year. A linear regression model was then fitted to assess the interannual trend in seasonal strength, with year as the independent variable and seasonal strength as the dependent variable.

### Spatial analysis

Given the large geographic area and heterogeneous population distribution of Beijing, we sought to determine whether the incidence of OIDD was randomly distributed or spatially clustered. Spatial analysis was therefore conducted to identify the location, type and temporal evolution of high-risk clusters. This information was critical for targeting region-specific interventions.

Using ArcGIS 10.8 software, a spatial analytical database for OIDD in Beijing was established by linking township/subdistrict-level incidence data with the vectorized electronic map of Beijing, using township/subdistrict names as the matching field. The geographic coordinate system used was WGS_1984, and the projected coordinate system was WGS_1984_UTM_Zone_50N.

Global spatial autocorrelation provides an overall characterization of spatial patterns across the study area. Moran’s *I* statistic was calculated to determine whether the spatial distribution of incidence exhibited clustering and to quantify the degree of clustering [6, 7]. Local spatial autocorrelation analysis was used to identify specific locations and types of spatial clusters based on the global results. After standardization, areas were categorized into five types: no significance, high-low, low-high, high-high, and low-low clusters [8].

Hot spot analysis was performed on the incidence of OIDD in Beijing using the Hot Spot Analysis tool in ArcGIS 10.8. This method computes the Getis-Ord Gi* statistic for each feature in the dataset. Based on the resulting z-scores and p-values, it identifies statistically significant spatial clusters of high values (hot spots) or low values (cold spots).

### Time-series modeling and forecasting

Descriptive and spatial analyses characterize historical patterns but cannot provide future projections. To support proactive public health planning, we employed a Seasonal Autoregressive Integrated Moving Average (SARIMA) model – a widely validated approach for infectious disease forecasting that explicitly models both trend and seasonality. This step translates retrospective epidemiological findings into operational predictions of case volumes and seasonal peaks.

The SARIMA model is a mathematical approach used to forecast future values of a time series by comprehensively analyzing its long-term trends, cyclical variations, and random fluctuations [9]. The model is generally denoted as SARIMA(p, d, q)(P, D, Q)ₛ, where “p” is the order of non-seasonal autoregression, “d” is the degree of non-seasonal differencing, “q” is the order of the non-seasonal moving average, “P” is the order of seasonal autoregression, “D” is the degree of seasonal differencing, “Q” is the order of the seasonal moving average, and “s” is the length of the seasonal cycle. As monthly incidence data were used in this study, “s” was set to 12.

Sequence stabilization: The “decompose” function in R (version 4.4.1) was used to generate a decomposition plot of the time series, visualizing trend, seasonal, and random components. Stationarity was assessed using the Augmented Dickey-Fuller (ADF) test. Non-stationary series were differenced to achieve stationarity [10].

Parameter Estimation and Model Selection: A grid search was conducted to traverse possible parameter combinations. The optimal model was selected by minimizing the Akaike Information Criterion (AIC). Parameters were estimated using the Maximum Likelihood Method (MLE) to ensure asymptotic efficiency of estimators [11, 12].

Residual Diagnosis and Model Validation: The Ljung-Box Q test was applied to assess autocorrelation in the residuals. A *P*-value > 0.05 indicated that the residuals constituted white noise, confirming that the model adequately captured all temporal dependencies.

Model Evaluation: Monthly case counts of OIDD in Beijing from January 2010 to December 2023 were used as the training set to construct the optimal SARIMA model. The training set encompassed 168 monthly data points, covering multiple full seasonal cycles and long-term trends to ensure robust model learning. Data from January to December 2024 served as the test set for model validation. This temporally split design directly simulates a real-world forecasting scenario by evaluating the model’s out-of-sample predictive performance on the most recent, unseen annual cycle. Performance was evaluated using the Mean Absolute Error (MAE), Root Mean Square Error (RMSE), Mean Absolute Percentage Error (MAPE), and Coefficient of Determination (*R*^2^) [13]. MAE is the average of absolute errors, which can better reflect the actual situation of prediction errors [14]. RMSE is widely used to measure the deviation between predicted values and actual values, with a lower bound of zero (indicating a perfect fit) and increasing infinitely as the model performance deteriorates. MAPE is an intuitive measure of relative error. MAPE ≤ 10% indicates high predictive accuracy; 10% < MAPE ≤ 20% suggests good accuracy; and 20% < MAPE ≤ 50% denotes acceptable prediction performance [15]. *R*^2^ reflects the degree of fitting between the predicted values and actual values, with a range of 0 to 1, where a value closer to 1 indicates a better fitting effect of the model.

Model Prediction: The finalized optimal model was used to forecast the monthly incidence of OIDD in Beijing from January to December 2025, along with the corresponding 80% Confidence Intervals (*CI*). The 80% *CI* is reported to provide a more focused and actionable range for the forecast. This can be interpreted as the model indicating a “most likely” scenario for future incidence, which is often more practical for initial public health planning and resource allocation, while still explicitly acknowledging the inherent uncertainty in the prediction.

### Statistical tools

Data collation and entry were performed using Microsoft Excel 2019. Graphical presentations were generated using R (version 4.4.1) and ArcGIS Spatial Statistics Toolbox (version 10.8). Data processing and modelling were conducted using the tseries, forecast and ggplot2 packages in R (version 4.4.1). A significance level of *α* = 0.05 was applied for all statistical tests.

## Results

### Epidemic profile

**Fig. 1:**
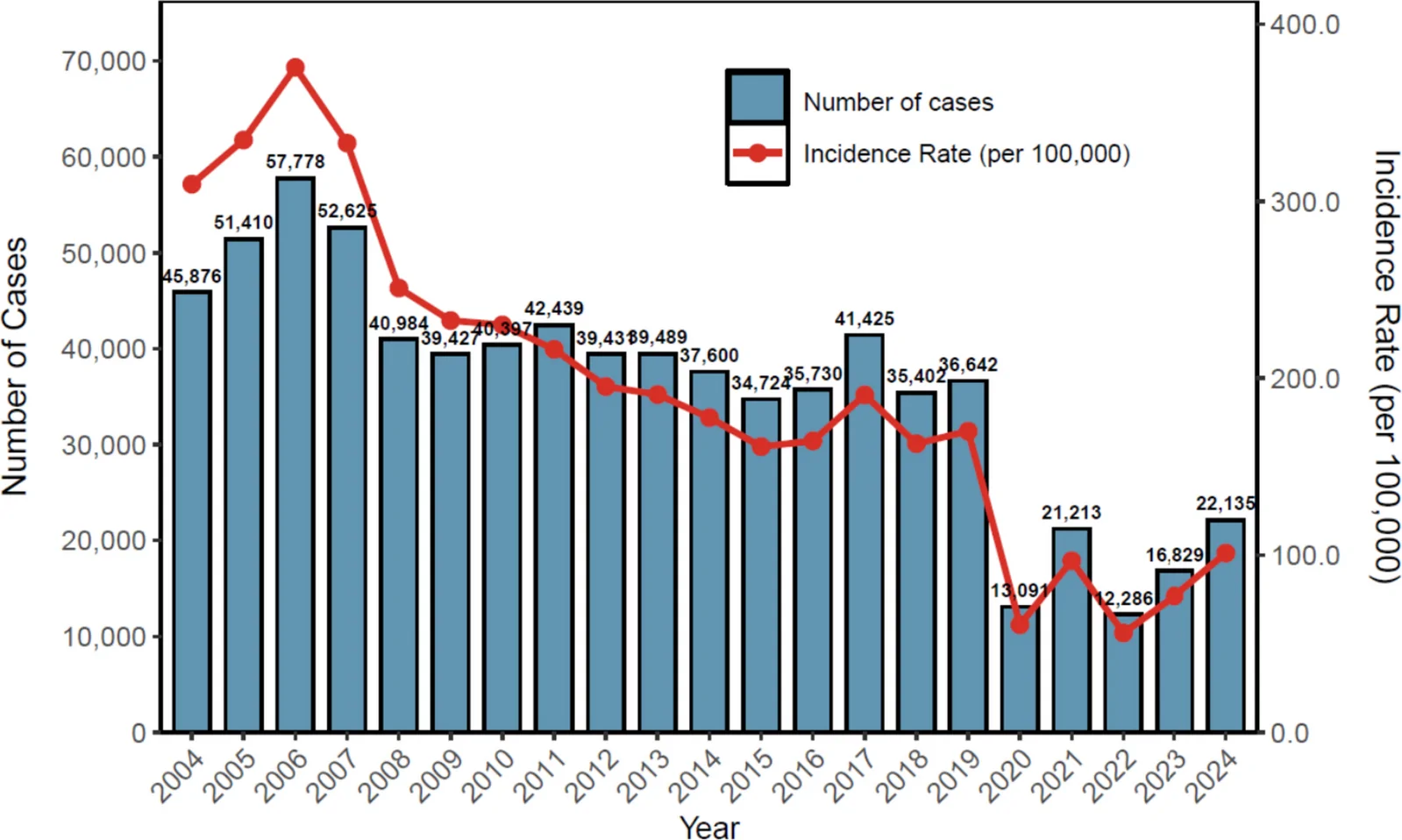
Number of cases and incidence rate of OIDD in Beijing from 2004 to 2024

Incidence exhibited an overall downward trend from 2006 to 2022, followed by a rebound from 2023 to 2024. A total of 756,933 cases of OIDD were reported in Beijing from 2004 to 2024. The overall incidence rate showed a significant downward trend (*χ*^2^ for trend = 139,682.972, *P* < 0.001). The highest annual incidence rate was recorded in 2006 (375.67 per 100,000 population), while the lowest was observed in 2022 (56.13 per 100,000). Following an initial rise, the annual incidence decreased to 161.39 per 100,000 in 2015. From 2016 to 2019, the incidence fluctuated slightly between 163 and 190 per 100,000, then declined to a near-low of 60.79 per 100,000 in 2020. A minor rebound occurred in 2021 (96.89 per 100,000), before the rate dropped to its lowest point in 2022. Subsequently, the incidence increased annually from 2022 to 2024 (Fig. 1).

During the 2004-2024 period, 25 fatal cases were reported, with 24 occurring between 2004 and 2016 and only one between 2017 and 2024 (in 2022). Among the deceased, 14 were male and 11 were female. These fatal cases were distributed across nine districts, with the highest number reported in Xicheng District (5 cases), followed by Fengtai and Mentougou Districts (4 cases each), Chaoyang and Shijingshan Districts (3 cases each), Dongcheng and Haidian Districts (2 cases each), and Daxing and Huairou Districts (1 case each). The age distribution of fatalities showed higher proportions in the younger and older age groups: 5 deaths (20.0%) occurred in individuals under 10 years old, 1 (4.0%) in the 10-49 age group, and 19 (76.0%) in those aged 50 years or older.

### Epidemiological characteristics: temporal, population and regional distribution

**Fig. 2:**
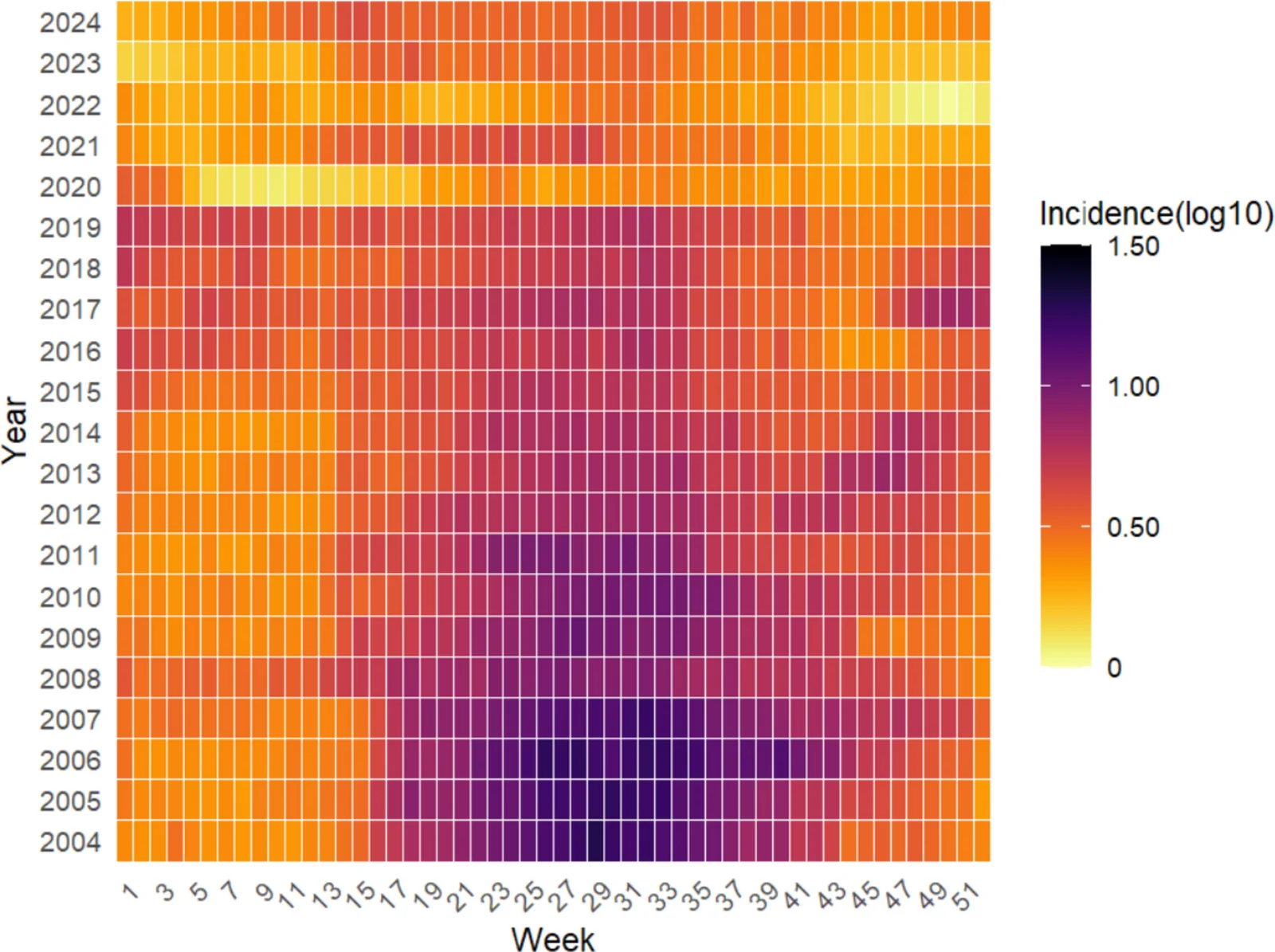
Heatmap of weekly incidence rates of OIDD in Beijing from 2004 to 2024

The seasonal pattern of OIDD in Beijing underwent changes between 2004 and 2024, characterized by shifting peak timings and a statistically significant weakening of seasonal intensity over time. Cases of OIDD were reported every month in Beijing between 2004 and 2024. From 2004 to 2014, the epidemiological pattern remained consistent, characterized by two annual peaks: a primary summer peak in July and August, and a secondary winter peak in November and December. Beginning in 2015, the seasonal characteristics shifted. Although the summer peak persisted from 2015 to 2022, the winter peak occurred later, primarily concentrated in December and January. Starting in 2023, the summer peak shifted earlier, mainly occurring in April and May (Fig. 2). Furthermore, the seasonal component of the incidence time series was extracted using a moving average-based decomposition method. The calculated seasonal intensity showed a statistically significant downward trend year by year (*β* = −1.003, 95% CI [−1.510, −0.496], SE = 0.258, *t* = −3.882, *P* < 0.001) (Table 1).

**Table 1:** The seasonal intensity distribution of the incidence of OIDD from 2004 to 2024

Year	Seasonal intensity	Year	Seasonal intensity	Year	Seasonal intensity
2004	31.31	2011	12.50	2018	15.76
2005	32.43	2012	11.74	2019	23.70
2006	35.49	2013	16.15	2020	5.46
2007	24.33	2014	11.76	2021	7.59
2008	4.31	2015	6.74	2022	6.72
2009	13.54	2016	16.92	2023	4.74
2010	15.17	2017	14.07	2024	7.15

A total of 386,812 male and 324,245 female cases were reported, with a male-to-female ratio of 1.19:1.00 from 2005 to 2024. Gender-specific incidence rates were presented in Table 2.

**Table 2:** The incidence rates of OIDD in Beijing by gender from 2005 to 2024

Year	Number of male cases (incidence rate per 100,000)	Number of female cases (incidence rate per 100,000)	Total number of cases (incidence rate per 100,000)
2005	28,966 (373.59)	22,444 (294.93)	51,410 (334.63)
2006	31,804 (408.23)	25,974 (342.24)	57,778 (375.67)
2007	29,340 (368.10)	23,285 (297.02)	52,625 (332.86)
2008	22,514 (273.60)	18,470 (227.98)	40,984 (250.97)
2009	21,307 (249.59)	18,120 (215.38)	39,427 (232.61)
2010	21,711 (245.74)	18,686 (214.41)	40,397 (230.18)
2011	22,744 (224.90)	19,695 (207.32)	42,439 (216.39)
2012	21,431 (205.93)	18,000 (184.07)	39,431 (195.34)
2013	21,377 (200.14)	18,112 (180.90)	39,489 (190.83)
2014	20,298 (186.10)	17,302 (168.95)	37,600 (177.79)
2015	18,699 (171.91)	16,025 (150.63)	34,724 (161.39)
2016	19,225 (172.67)	16,505 (156.13)	35,730 (164.62)
2017	22,406 (201.35)	19,019 (179.41)	41,425 (190.64)
2018	19,107 (172.54)	16,295 (153.25)	35,402 (163.09)
2019	19,762 (180.36)	16,880 (159.47)	36,642 (170.10)
2020	7070 (64.62)	6021 (56.83)	13,091 (60.79)
2021	11,324 (101.15)	9889 (92.44)	21,213 (96.89)
2022	6622 (59.23)	5664 (52.89)	12,286 (56.13)
2023	8997 (80.73)	7832 (73.22)	16,829 (77.05)
2024	12,108 (108.80)	10,027 (93.44)	22,135 (101.26)
Total	386,812 (202.46)	324,245 (175.05)	711,057 (188.96)

Due to incomplete records of sex-specific case counts and incidence rates in the China Information System for Disease Control and Prevention for 2004, data are presented from 2005 onwards

**Fig. 3:**
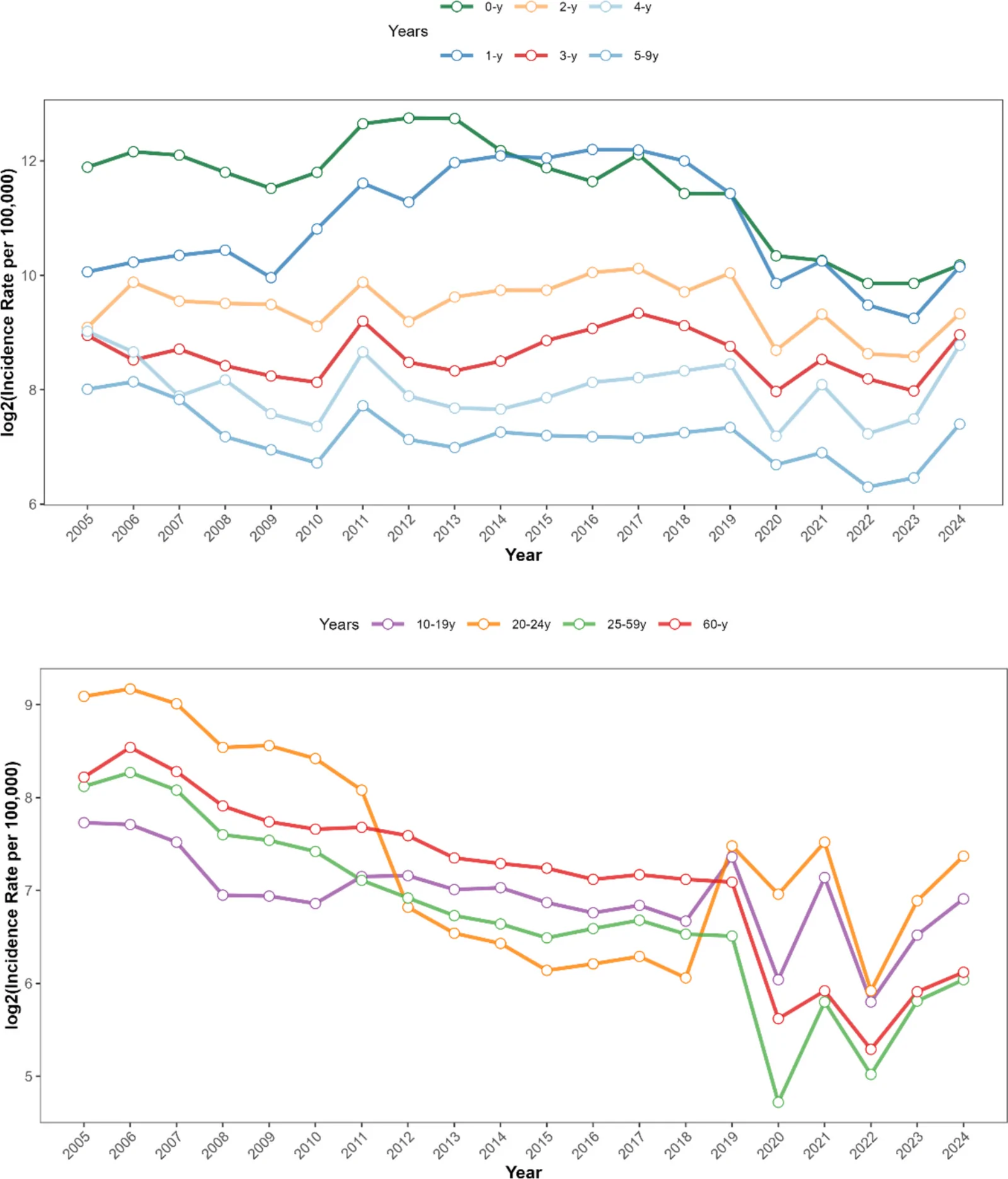
The temporal distribution of the incidence of OIDD among different age groups in Beijing from 2005 to 2024. Note: Due to incomplete records of age-specific incidence data in the China Information System for Disease Control and Prevention for 2004, the figure presents data from 2005 onward

Substantial differences in incidence rates were observed across age groups over the 20-year period. To examine temporal trends, log-transformed values of incidence rates were analyzed. The under-1 year age group showed the highest average annual incidence rate (3502.75 per 100,000), with high-level incidence fluctuations between 2005 and 2014 followed by a rapid decline thereafter. The 1- age group showed a broader and more gradual peak from 2013 to 2019, with both a later and lower peak incidence compared with the under-1 year age group. Although the overall incidence was higher in the under-1 year age group, it was surpassed by the 1- group between 2015 and 2019. Incidence levels in the 2-9 age group remained relatively stable. The 10-19, 20-24, 25-59, and ≥ 60 age groups exhibited similar trends, all showing a gradual decline from 2005 to 2018. Between 2019 and 2024, the log-transformed incidence rates for all age groups exhibited a “W”-shaped pattern, with two distinct troughs occurring in 2020 and between 2022-2023. A marked increase in log-transformed incidence was observed across all age groups after 2023 (Fig. 3).

The five categories with the highest reported case numbers were children in home-based care (212,393 cases, 28.06%), office workers (116,431 cases, 15.38%), retirees (95,458 cases, 12.61%), homemakers and unemployed individuals (83,547 cases, 11.04%), and students (79,944 cases, 10.56%).

**Fig. 4:**
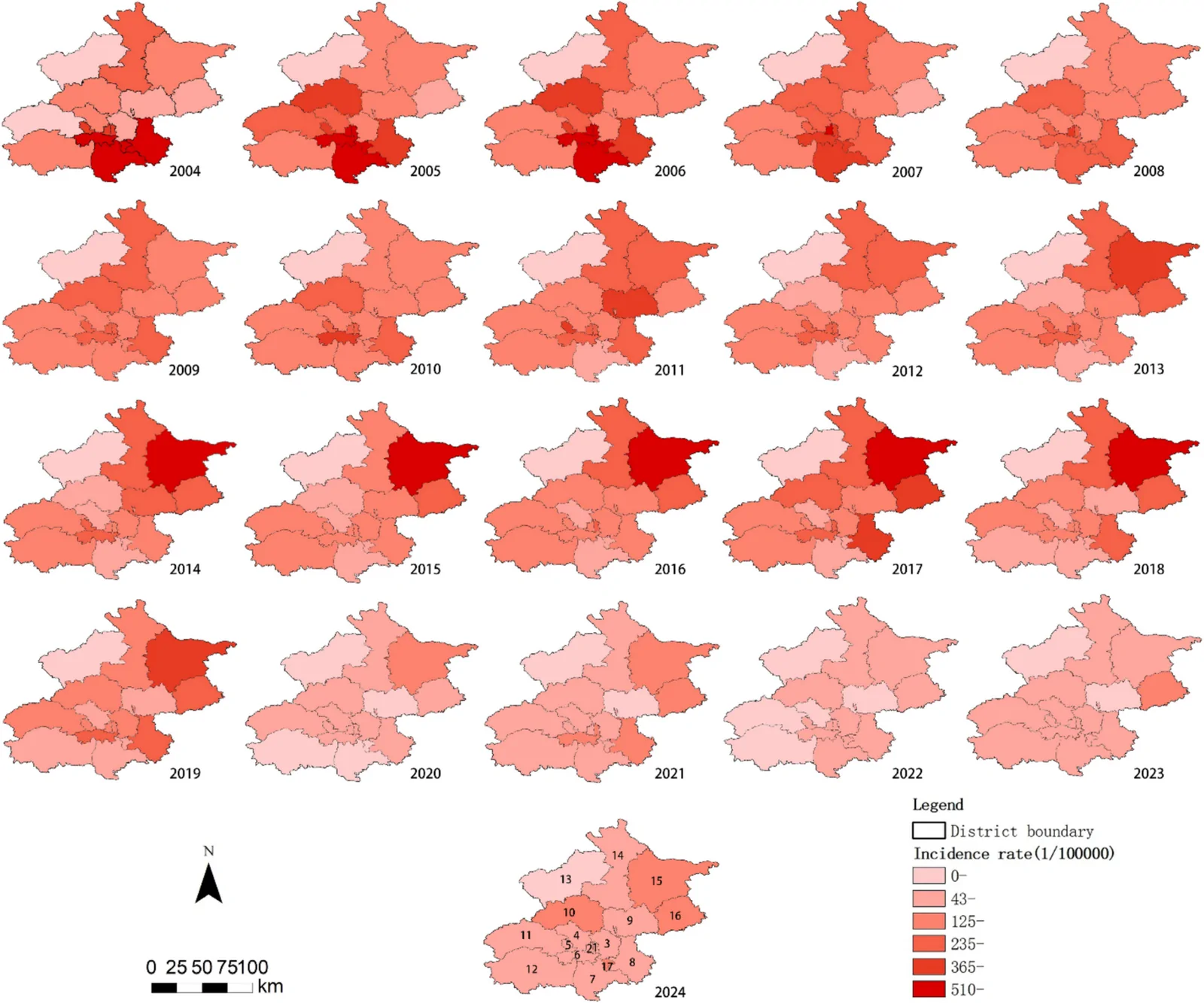
The spatial distribution of the incidence rate of OIDD in Beijing from 2004 to 2024. (1: Dongcheng District; 2: Xicheng District; 3: Chaoyang District; 4: Haidian District; 5: Shijingshan District; 6: Fengtai District; 7: Daxing District; 8: Tongzhou District; 9: Shunyi District; 10: Changping District; 11: Mentougou District; 12: Fangshan District; 13: Yanqing District; 14: Huairou District; 15: Miyun District; 16: Pinggu District; 17: Jingkai District)

Cases of OIDD were reported in all 17 districts of Beijing from 2004 to 2024. The city’s average annual incidence rate from 2004 to 2019 was 222.28 per 100,000 population. The top five districts with the highest average annual incidence rates during this period were Miyun District (349.27/100,000), Xicheng District (337.85/100,000), Fengtai District (325.44/100,000), Dongcheng District (322.10/100,000), and Tongzhou District (298.66/100,000). Yanqing District recorded the lowest average annual incidence rate (32.28/100,000). Between 2004 and 2010, higher incidence rates were observed in several urban districts (Dongcheng, Xicheng, Shijingshan, and Fengtai) and southeastern suburban districts (Daxing and Tongzhou). Changping District also experienced relatively high incidence rates during 2005-2006. From 2011 to 2016, high incidence rates shifted to northeastern suburban districts, primarily Shunyi, Huairou, Miyun, and Pinggu Districts. A resurgence in incidence was noted in Tongzhou District between 2017 and 2019. During 2020-2024, incidence rates declined across all districts, with a citywide average annual incidence of 78.48 per 100,000 population. The top five districts by average annual incidence in this recent period were Miyun District (145.76/100,000), Pinggu District (144.31/100,000), Fengtai District (106.41/100,000), Huairou District (106.27/100,000), and Tongzhou District (105.01/100,000). Yanqing District again reported the lowest rate (19.19/100,000) (Fig. 4).

### Analysis of spatiotemporal variation patterns

A global spatial autocorrelation analysis was performed on the annual district-level incidence data. The results indicated the presence of positive spatial autocorrelation in each year, with Moran’s *I* values ranging from 0.18 to 0.44 and associated *Z*-scores between 5.74 and 13.13 (all *P* < 0.01). These findings confirmed that the distribution of OIDD cases in Beijing exhibited significant spatial clustering throughout the 2005-2024 period (Supplementary Table S1).

**Fig. 5:**
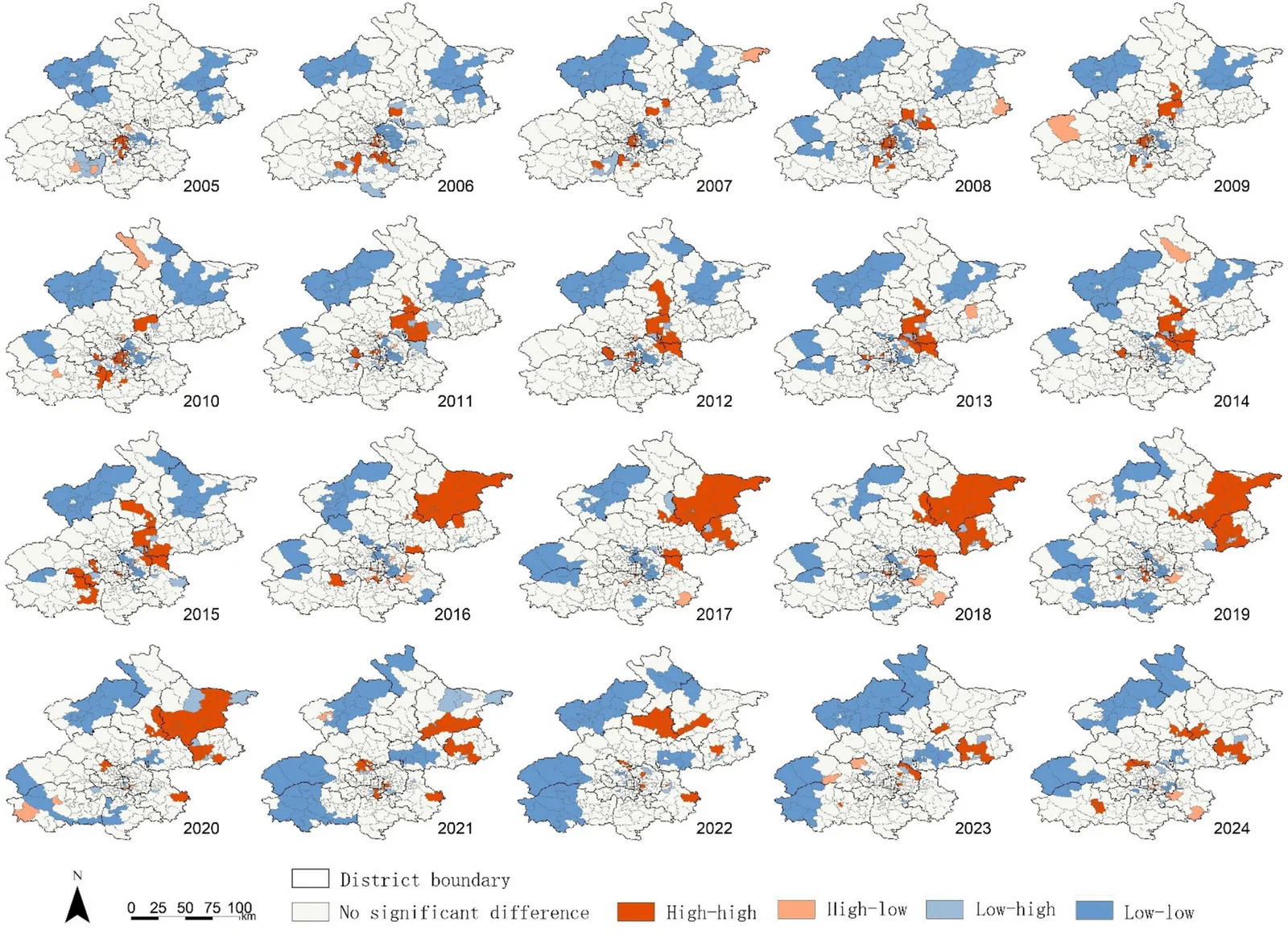
Local spatial autocorrelation analysis of OIDD in Beijing from 2005 to 2024. Note: Due to incomplete records of patient residential address data in the China Information System for Disease Control and Prevention for 2004, the figure presents data from 2005 onward

Results from the local spatial autocorrelation analysis revealed distinct regional heterogeneity in the spatiotemporal patterns of OIDD incidence in Beijing from 2005 to 2024. The spatial clustering patterns were categorized into four types: high-high, high-low, low-high, and low-low clusters. In 2005, high-high clusters were predominantly located in the central urban districts, including Dongcheng, Xicheng, and Fengtai. From 2006 to 2010, additional high-high clusters emerged in western Shunyi, eastern Fangshan, and northern Daxing Districts. Beginning in 2011, the number of high-high clusters within core urban districts decreased, and the clusters gradually expanded toward suburban and exurban regions. Between 2011 and 2015, high-high clusters were mainly concentrated in Tongzhou, Shunyi, and Huairou Districts. From 2016 to 2020, the spatial focus of high-high clusters shifted northeastward, primarily encompassing Miyun and Pinggu Districts. During 2021-2024, high-high clusters became more dispersed and exhibited a declining trend in number. Throughout the entire study period (2005-2024), persistent low-low clusters were consistently observed in Yanqing District, northern Huairou, western Mentougou, and western Fangshan Districts (Fig. 5).

**Fig. 6:**
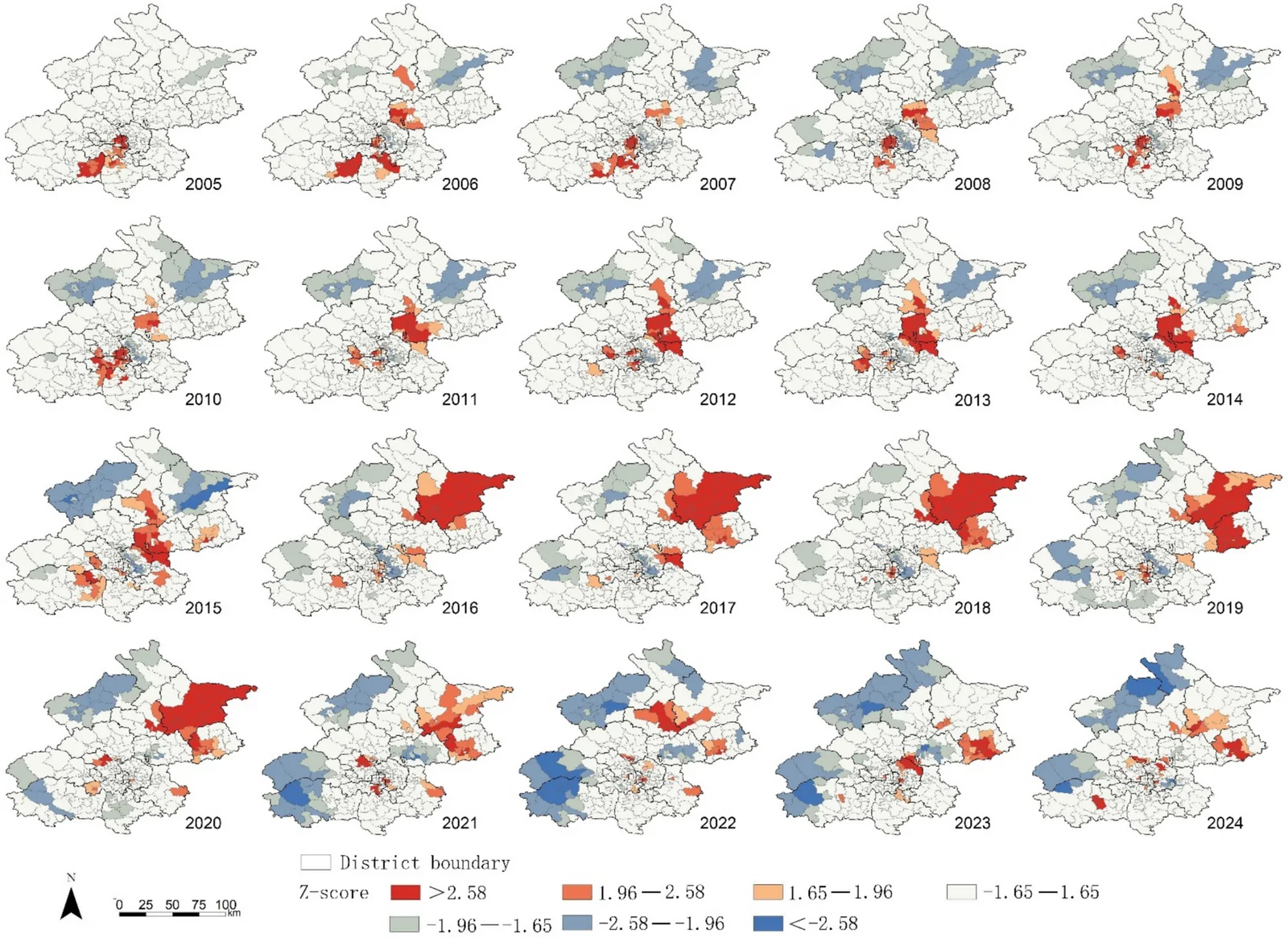
Analysis of hotspots of OIDD in Beijing from 2005 to 2024. Note: Due to incomplete records of patient residential address data in the China Information System for Disease Control and Prevention for 2004, the figure presents data from 2005 onward

Hot spot analysis revealed that in 2005, hot spots of OIDD in Beijing were mainly concentrated in Dongcheng District, Xicheng District, eastern Fengtai District, eastern Fangshan District, and northwestern Daxing District. From 2006 to 2010, hot spots also emerged in western Shunyi District and southern Huairou District. Beginning in 2011, the number of hot spots in the central urban districts decreased, while those in the eastern suburban areas increased. Between 2011 and 2015, hot spots were primarily located in northern Tongzhou District, western Shunyi District, and southern Huairou District. From 2016 to 2020, the hot spot regions shifted toward the northeastern exurban areas of Beijing, predominantly clustering in Miyun District and Pinggu District. During 2021-2024, hot spots became more dispersed and decreased in number (Fig. 6).

### SARIMA model for incidence trend prediction

To better visualize the incidence trend and seasonal patterns and to enhance the robustness of the modeling analysis, the original monthly time series data were preprocessed using a natural logarithm transformation. A time series plot of the natural logarithm of monthly reported cases of OIDD in Beijing from January 2010 to December 2024 was constructed to observe long-term trends, seasonal variations, and random fluctuations. The results indicated that the incidence exhibits periodic fluctuations and represents a non-stationary series **(**Supplementary Fig. S1).

Upon examining the Autocorrelation Function (ACF) and Partial Autocorrelation Function (PACF) plots of the original series and considering the distinct seasonal periodicity in the data, first-order seasonal differencing and first-order non-seasonal differencing were applied to remove non-stationary trends and obtain a stationary series. The ACF and PACF plots of the differenced series were then analyzed. The ADF test yielded a test statistic of *t* = −6.410 (*P* < 0.01), confirming that the differenced time series was stationary and suitable for constructing a SARIMA model. The model parameters were selected as d = 1 and *D* = 1 **(**Supplementary Fig. S2).

Following systematic evaluation and fitting of parameters p, q, *P*, and *Q*, the optimal predictive model was identified as SARIMA (1,1,1) (1,1,1) ₁₂, with an AIC value of 95.7. The reported case numbers from January to December 2024 were used as the test set to compare the actual case count against the model-predicted values, thereby validating its predictive performance. The results demonstrated that the optimal SARIMA (1,1,1) (1,1,1) ₁₂ model yielded the following performance metrics: MAE = 272.33, RMSE = 308.12, MAPE = 17.46%, and* R*^2^ = 0.62 (Table 3).

**Table 3:** Comparison of candidate SARIMA models

Models	AIC	MAE	RMSE	MAPE(%)	R^2^
SARIMA(1,1,1)(1,1,1)_12_	95.7	272.327	308.124	17.46	0.623
SARIMA(2,1,0)(0,1,1)_12_	95.7	263.754	339.979	17.54	0.541
SARIMA(0,1,1)(1,1,1)_12_	97.4	276.344	311.808	17.66	0.614
SARIMA(1,1,0)(1,1,1)_12_	97.7	277.120	312.547	17.72	0.612
SARIMA(1,1,1)(0,1,1)_12_	98.5	312.439	372.109	20.46	0.451
SARIMA(0,1,1)(0,1,1)_12_	99.3	317.569	376.311	20.86	0.438
SARIMA(1,1,0)(0,1,1)_12_	99.5	317.030	376.504	20.83	0.438
SARIMA(0,1,2)(1,1,0)_12_	103.2	324.810	429.244	20.58	0.269
SARIMA(2,1,0)(1,1,0)_12_	106.6	293.245	422.176	19.54	0.293
SARIMA(0,1,1)(1,1,0)_12_	109.7	291.671	420.864	19.97	0.297

A Ljung-Box Q test was performed on the residuals of the SARIMA (1,1,1) (1,1,1) ₁₂ model. The results indicated that the residuals constituted white noise (*χ*^2^ = 21.349, *P* = 0.618) (Supplementary Fig. S3).

**Fig. 7:**
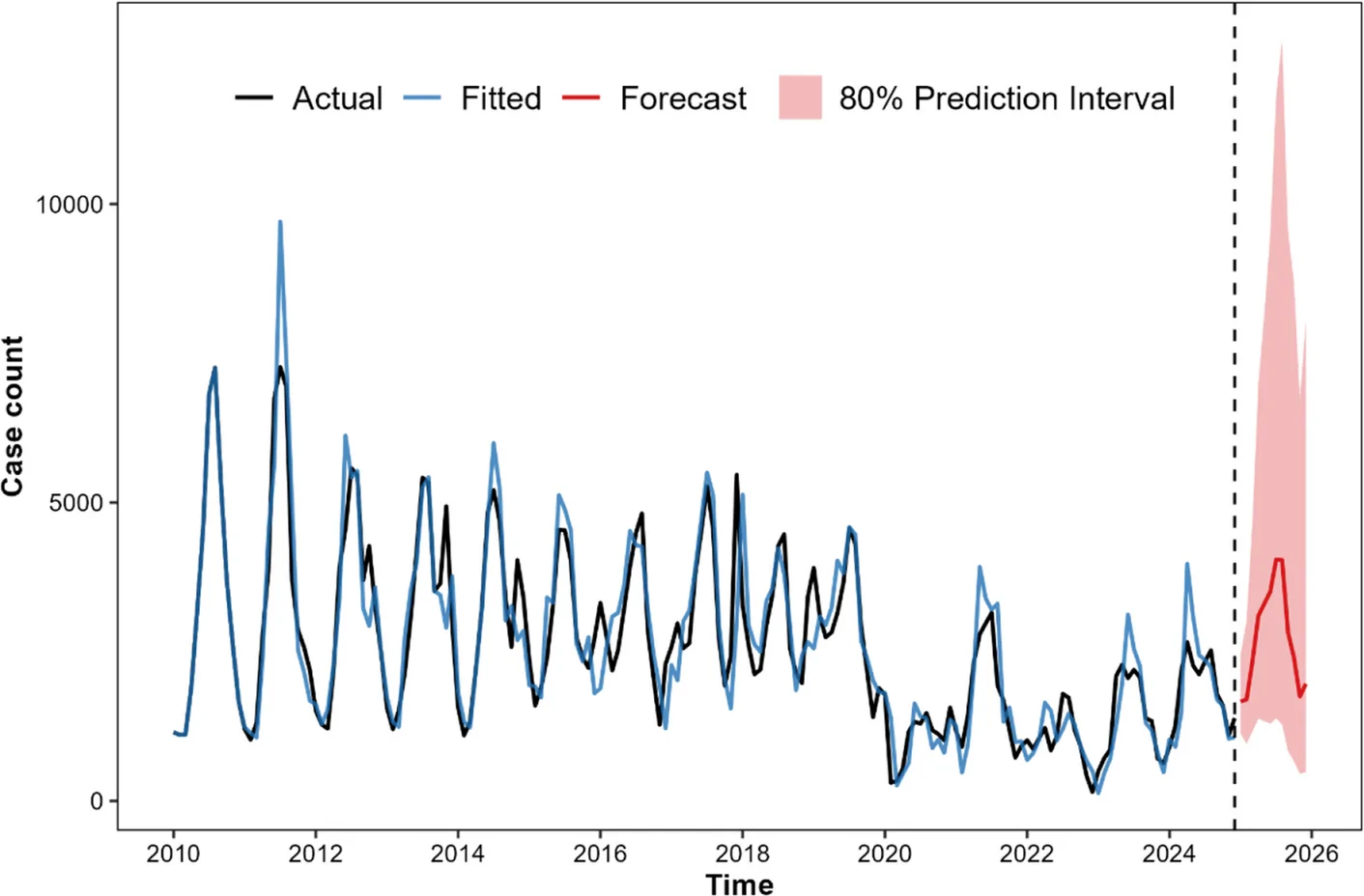
Prediction results of monthly cases of OIDD in Beijing

The fitted model was used to extrapolate and generate monthly case estimates and associated 80% *CI* for OIDD in Beijing for 2025 **(**Supplementary Table S2). The predicted total number of cases for 2025 was 32,638, which is projected to increase by approximately 47% compared to 2024. The case count pattern is projected to exhibit a bimodal pattern, with a primary peak occurring from June to August and a secondary peak between December 2025 and January 2026 (Fig. 7).

## Discussion

The reported incidence of OIDD in Beijing showed a significant increase from 2004 to 2006. This upward trend may be attributed to the gradual maturation of the China Disease Prevention and Control Information System during its initial implementation phase, coupled with improved awareness among healthcare providers regarding infectious disease reporting and enhanced overall surveillance quality. From 2007 to 2020, the number of reported cases generally exhibited a sustained downward trend, likely associated with the initial effectiveness of health education campaigns conducted by disease control authorities and improved public hygiene awareness.

During 2021-2022, when comprehensive Non-Pharmaceutical Interventions (NPIs)—such as mask-wearing, frequent hand hygiene, regular ventilation, and maintaining safe social distancing—were implemented across society, Beijing experienced a low incidence period for OIDD. However, following the downgraded management of COVID-19 to Class B on January 8, 2023, the city saw a significant rebound in cases, consistent with trends in other Chinese regions [16, 17]. This resurgence may be related to three factors: first, relaxation of NPIs, which may potentially increase the risk of enteric infectious disease transmission; second, low transmission during 2021-2022, possibly leading to waning immunity and accumulation of susceptible individuals; and third, increased frequency of high temperatures and extreme precipitation events in recent years, which has been associated with diarrheal disease incidence [18–21].

OIDD in Beijing exhibits a bimodal seasonal distribution, with peaks in summer and winter. Our data indicated that winter resurgence coincides with major population movement events, underscoring the influence of local sociocultural factors. This observed pattern aligns with historical trends reported nationally, as well as in Anhui Province and Beijing itself [4, 5, 22, 23]. High temperatures have been shown to increase the risk of other infectious diarrhea. A study by Zhou et al. [24] indicated that a 1 °C increase in the 6-day moving average of temperature was associated with a 2.68% (95% *CI*: 1.83-3.52) rise in outpatient visits for infectious diarrhea in Shanghai, China. Similarly, research in South Africa demonstrated that a 5 °C increase in weekly maximum temperature was linked to a 32% (95% *CI*: 1.22-1.41) increase in diarrheal cases [25]. The underlying mechanism may involve accelerated food spoilage and enhanced survival and reproduction of pathogens under high temperatures, facilitating outbreaks of bacterial diarrhea [21, 26, 27]. Thus, during summer months, timely cooling measures and strengthened hygiene management of food and water sources are recommended.

However, low temperatures can also trigger sharp outbreaks of diarrhea. D’Souza et al*.* [28] suggested that colder winter conditions may promote the survival and transmission of viruses, particularly norovirus. This study also found that the summer peak in 2020 was lower than in previous years. This anomaly may be attributed to the COVID-19 outbreak at the Xinfadi Market in June 2020, which prompted school closures and reduced population aggregation in Beijing, thereby decreasing opportunities for disease transmission and potentially lowering healthcare-seeking behavior [22].

Similar to findings in Jiangsu and Yunnan, we observed the highest incidence among children aged 0-2 years, reinforcing the importance of early-life hygiene interventions [4, 29–31]. This elevated susceptibility is primarily attributed to delayed vaccination, an immature immune system, underdeveloped hygiene habits, and increased contamination risk of feeding utensils. Furthermore, sample surveys conducted in Guangdong, Henan, and Gansu provinces revealed that young children had the highest medical consultation rate (79.9%) for diarrheal diseases, suggesting that higher healthcare-seeking behavior also contributes to the elevated reported incidence in this age group [32–34]. These findings underscore that infectious diarrhea in children warrants attention as a significant public health issue [35].

Higher incidence in males may be linked to more frequent social activities and dining out, leading to greater exposure [30, 36]. Our study uniquely demonstrated that high-high clustering areas shifted from central urban districts to suburban districts between 2005 and 2024. These findings suggest that suburban regions should be prioritized for health education and epidemic control interventions. In contrast, “low-low” clusters have consistently been observed in Yanqing District, northern Huairou, western Mentougou, and western Fangshan. The persistently low reported incidence in these areas may be partly explained by their remote mountainous nature, potentially leading to low healthcare-seeking behavior among residents and insufficient diagnostic awareness among local health workers. Thus, the actual number of cases may be underreported. Overall, the hot spot analysis of incidence trends from 2005 to 2024 aligns well with the patterns identified through local spatial autocorrelation analysis.

The SARIMA model, an extension of the ARIMA model incorporating seasonal differencing components, offers enhanced capability for seasonal adjustment. OIDD is influenced by multiple factors and exhibits distinct periodicity and seasonality [37].

A prerequisite for SARIMA modeling is a time series length exceeding 30 data points. This study utilized incidence data spanning 180 months from 2010 to 2024, encompassing 15 complete seasonal cycles, which fully satisfies the data volume requirements for model construction.

The optimal model selected was SARIMA (1,1,1) (1,1,1) ₁₂. The final model selection was based on superior overall predictive performance. Although another candidate model, SARIMA (2,1,0) (0,1,1) ₁₂, shared an identical AIC value, the SARIMA (1,1,1) (1,1,1) ₁₂ model was chosen for its lower prediction error (RMSE, MAPE) and higher explanatory power (*R*^2^) on the test set. Evaluation on the test set yielded a MAPE of 17.46%, indicating the model provides relatively accurate predictions. While this moderate prediction error reflects the model’s inherent limitation in incorporating influential external factors (e.g., vaccination, meteorology), the accuracy is acceptable for public health purposes, as it reliably forecasts the timing and scale of seasonal epidemics to inform preparedness and resource planning. Notably, when presenting the 80% CI for the 2025 forecasts, some uncertainty still exists beyond this reported interval, as inherent uncertainties in time series forecasting may lead to slight fluctuations in actual incidence. The forecasting results of this study indicated that the number of cases in Beijing in 2025 is projected to increase by approximately 47% compared to 2024. The annual incidence pattern is expected to maintain its characteristic bimodal distribution, with peaks occurring in summer and winter, consistent with previous years.

Overall, this study provides the first long-term, high-resolution spatiotemporal analysis of OIDD in Beijing, revealing both temporal rebound trends and spatial shifts of high-risk areas, with implications for targeted disease control in megacities.

This study has several limitations. First, the data were obtained from the China Disease Prevention and Control Information System, which is a passive surveillance system. As such, the data are subject to reporting bias, as some affected individuals may not seek medical care due to personal reasons, potentially leading to an underestimation of the true disease burden. Additionally, variability in diagnostic confirmation across healthcare settings introduces further bias, as differences in diagnostic practices across facilities may lead to case misclassification and reduced data accuracy. Second, the SARIMA model does not incorporate external variables such as meteorological conditions, economic factors and vaccination coverage, and drastic changes in these factors may cause significant prediction deviations. Third, this study was conducted in Beijing—a densely populated megacity with unique socioeconomic and infrastructural characteristics—and thus the findings may not be directly generalizable to rural or less densely populated settings. Similar analyses in other regions are needed to validate the broader applicability of these results. Fourth, future work should integrate OD matrices or township-level census data to disentangle the relative contributions of residential density, workplace aggregation, and healthcare-seeking migration to the formation of suburban hotspots.

## Conclusions

In summary, the incidence of OIDD in Beijing (2004-2024) showed an initial decline followed by a recent rebound. This first spatiotemporal analysis of a megacity reveals a distinct shift of high-high clusters from central urban to suburban areas. SARIMA model predictions point to continued increases in 2025. These findings underscore the need to monitor shifting transmission foci, target interventions during seasonal peaks, promote hygiene practices among caregivers of children aged 0-2 years, and strengthen water and food safety supervision. Such precision-based, dynamic strategies are vital to curb resurgence in megacities and provide a reference for other densely populated regions.

## Supplementary Information

Supplementary Material 1. Supplementary Fig. S1: Time series decomposition chart of the number of cases of OIDD in Beijing from 2010 to 2024. Supplementary Fig. S2: ACF and PACF plots of the natural logarithm of the case count of OIDD in Beijing from 2010 to 2024. (A) plots before the difference. (B) plots after one-order seasonal difference and one-order non-seasonal difference. Supplementary Fig. S3: ACF plot of the residuals of the SARIMA (1,1,1) (1,1,1)12 model.

## Data Availability

The datasets used during the current study are available from the corresponding author on reasonable request. The datasets used during the current study are available from the corresponding author on reasonable request.

## Abbreviations

ACF: Autocorrelation Function
ADF: Augmented Dickey-Fuller
AIC: Akaike Information Criterion
CI: Confidence Intervals
MAE: Mean Absolute Error
MAPE: Mean Absolute Percentage Error
MLE: Maximum Likelihood Method
NPIs: Non-Pharmaceutical Interventions
OIDD: Other Infectious Diarrheal Diseases
PACF: Partial Autocorrelation Function
*R*^2^: Coefficient Of Determination
RMSE: Root Mean Square Error
SARIMA: Seasonal Autoregressive Integrated Moving Average
SE: Standard Errors
