# Supplementary material for: Spatiotemporal patterns and 2025 forecasting of other infectious diarrheal diseases in Beijing, China: a 21-year population-based study (2004-2024)
Source: BMC Public Health. 2026 Jun 4;26:2768. doi: 10.1186/s12889-026-27967-0 (PMC13637398; doi:10.1186/s12889-026-27967-0)
Supplement: Supplementary file 1 [file 12889_2026_27967_MOESM1_ESM.docx]

Supplementary Fig. S1: Time series decomposition chart of the number of cases of OIDD in Beijing from 2010 to 2024

Supplementary Fig. S2: ACF and PACF plots of the natural logarithm of the case count of OIDD in Beijing from 2010 to 2024. (A) plots before the difference. (B) plots after one-order seasonal difference and one-order non-seasonal difference.

Supplementary Fig. S3: ACF plot of the residuals of the SARIMA (1,1,1) (1,1,1)12 model
